# Integrated assessment of thiram toxicity in pangasius fish: Erythrocytic abnormalities, gill and intestinal defense mechanisms, and genotoxicity

**DOI:** 10.1371/journal.pone.0353763

**Published:** 2026-07-15

**Authors:** Nimra Sageer, Areeb Nawaz, Riaz Hussain, Ghulam Mustafa, Laila A. AL Essa, Khalid M. Alsyaad, Ahmed Aljazzar, Yehia Hazzazi, Mari Sumayli, Sarmad Rehan, Khalid Mehmood

**Affiliations:** 1 Department of Zoology, The Islamia University of Bahawalpur, Bahawalpur, Pakistan; 2 Department of Pathology, Faculty of Veterinary and Animal Sciences, The Islamia University of Bahawalpur, Bahawalpur, Pakistan; 3 Department of Mathematical Sciences, College of Science, Princess Nourah bint Abdulrahman University, Riyadh, Saudi Arabia; 4 Department of Biology, College of Science, King Khalid University, Abha, Saudi Arabia; 5 Department of Pathology, College of Veterinary Medicine, King Faisal University, Hofuf, Al-Ahsa, Saudi Arabia; 6 Department of Biology, College of Science, Jazan University, Jazan, Kingdom of Saudi Arabia; 7 Department of Biology, College of Science, Jazan University, Jazan, Saudi Arabia; 8 Department of Anatomy, University of Agriculture, Faisalabad, Pakistan; 9 Department of Clinical Medicine and Surgery, Faculty of Veterinary and Animal Sciences, The Islamia University of Bahawalpur, Bahawalpur, Pakistan; Sindh Agriculture University, PAKISTAN

## Abstract

Pesticides play a crucial role in agriculture, public health management, and veterinary practice to control pests. However, the persistent use of pesticides poses a significant threat to aquatic ecosystems and non-target organisms, such as fish. The genotoxicity, oxidative stress, and erythrocytic toxicity were assessed in fish exposed to different sublethal concentrations (5, 10, and 15 µg/L) of thiram for 12 days. Blood and other tissues (gills and intestine) were obtained from each fish reared in each group at days 4, 8, and 12 of the experiment. Thin blood film/smear examination indicated significantly (*P ≤ 0.05*) increased morphological and nuclear changes in erythrocytes, such as the formation of micronuclei, notched nuclei, lobed nuclei, and tear-shaped erythrocytes in fish exposed to higher doses of thiram. The contents of antioxidant enzymes such as catalase (CAT), reduced glutathione (GSH), peroxidase (POD), and superoxide dismutase (SOD) were significantly (*P ≤ 0.05*) decreased and the oxidative stress biomarkers including reactive oxygen species (ROS) and thiobarbituric acid reactive substances (TBARS) were significantly (*P ≤ 0.05*) increased in gills and intestinal tissues of exposed fish. The results on genotoxicity determined using the comet assay technique revealed significantly (*P ≤ 0.05*) high levels of genomic instability in terms of DNA damage in isolated cells of the gills and intestine of fish reared in water containing higher concentrations of thiram. At the microscopic level, various sections of the gills of fish treated with higher doses of thiram unveiled moderate to severe histopathological ailments such as degeneration of cartilaginous core, degeneration and disruption of primary and secondary lamellae of gills, necrosis of lamellar epithelial cells, telangiectasia, and curling of secondary lamellae. The findings of this study indicated that extremely low doses of thiram lead to the induction of adverse effects in freshwater fish. Hence, there is a need to develop suitable control measures regarding the use of environmentally friendly chemicals and for the protection of aquatic environments.

## Introduction

Pangasius or catfish is one of the well-recognized freshwater species of global importance in aquaculture because of their adaptability and economic value [1,2]. It has been recorded that Pangasius fish is distributed in approximately 40 countries in freshwater habitats like canals, rivers, and lakes throughout the world, while juveniles of Pangasius fish are marketed as a preferred aquarium fish [3]. Moreover, studies have indicated that Pangasius fish is known as one of the most economically important among various freshwater fish species reared across the globe worldwide [3]. Being a valuable source of micronutrients, essential fatty acids, and proteins, Pangasius fish is considered as an important source of a healthy diet [4]. However, freshwater fish species are incredibly susceptible to a variety of environmental pollutants, including pesticides/industrial wastes, and agricultural runoff. Pangasius fish, being a freshwater species, is extensively involved in global aquaculture, which can be susceptible to pesticides. Several studies have highlighted the importance and emphasized the need for the development of effective techniques to control environmental pollution for sustainable aquaculture practices [5]. The extensive use of pesticides in modern agriculture, veterinary practices, and public health management has resulted in contamination of various aquatic ecosystems, causing serious concerns regarding their deleterious effects on non-target organisms, especially fish [6–8]. Moreover, it is highlighted that pesticides induce toxicity via disruption of normal physiological mechanisms (redox homeostasis) in terms of depletion of antioxidant enzymes (CAT, T-AOC, GSH, and SOD), upregulation of signaling pathways (HIF-1/VEGF, Rap1-PI3K/AKT, and Ras-MAPK) in exposed organisms [9]. Among various pesticides and fungicides, dithiocarbamate (DTC) fungicides have been frequently used since the 1940s to control fungal infections in ornamental and food crops as well as in public health management [10]. Among different DTC fungicides, thiram induces adverse toxic effects on aquatic organisms and wildlife [11,12]. Studies have reported that the frequent use of these synthetic chemicals regularly and without following the recommended guidelines of the manufacturer causes them to enter water bodies and can induce major damage to aquatic life, alteration of normal functions of aquatic ecosystems, leading to decline of biodiversity [13–15]. Thiram is commonly, persistently, and widely used in agriculture for the protection of fruits, vegetables, and ornamental plants globally, with about 140 tons per year in the US and about 150 tons in South Korea during the 2013–2016 period [16,17]. It is recorded that thiram can persist in water (0.27 to 2.52 mg/L) following its applications, which can potentially harm aquatic organisms [18].

Thiram has been linked to inducing thyroid dysfunctions and liver toxicity [16]. Reports have indicated that exposure to various environmental contaminants, including pesticides, insecticides, and herbicides [19], induces different structural abnormalities in liver and brain tissues of exposed organisms, leading to inflammatory responses and physiological disorders [20–22]. Different abnormalities, such as abnormal cleft palates, spinal deformities, and curled ribs, were examined in fetuses of rats that were exposed to thiram [23]. In addition, thiram is reported to trigger hemato-biochemical, histopathological, oxidative, and reproductive defects in albino rats [24]. Thiram leads to the induction of oxidative stress and tissue damage in Daphnia magna [25]. Studies have reported reproductive toxicity of thiram in zebra fish (Danio rerio), and the developmental defects of embryos, craniofacial and spinal curvature, as well as thyroid dysfunction [26,27].

Oxidative stress induces rapid over-generation of reactive oxygen species (ROS) that lead to depletion of antioxidant defense mechanisms in exposed organisms [28,29]. Such an imbalance leads to cell damage that subsequently destroys important cellular components, such as lipids, proteins, and DNA [30,31]. The destruction of blood cells and tissues will also create more cytotoxicity as a result of pesticides. Various environmental pollutants, such as biofertilizers, heavy metals, and pesticides. industrial effluents and a combination of these compounds greatly influence the well-being of aquatic life and lead to serious consequences, including a reduced efficiency of reproductive functions, low growth rates, and/or death [32,33]. Exposure to thiram in freshwater fish (*Labeo rohita*) induces genotoxicity, oxidative stress, and cytotoxicity [34]. It has been documented that exposure to different synthetic compounds can cause genotoxic effects such as genetic damage, mutation, and chromosomal aberration [35]. In previous published literature, various reports are available regarding the toxic effects of thiram in albino rats [24], at higher concentrations (50 mg/kg and 75 mg/kg), genotoxicity in *labeo rohita* exposed to 80 μg/L and 120 μg/L of thiram [34], chickens treated with 50 mg/kg of thiram [36,37] induction of oxidative stress in *Daphnia magna* [21] treated with thiram (0.05–0.50 mg/L), developmental toxicity in zebra fish [17] exposed to thiram (8.87–13.10 μg/L), intestinal and nephrotoxicity in rats [38], oxidative stress, genotoxicity and histopathological ailments in gills of common carp treated with higher doses (80 and 120 μg/L of thiram [39]. Furthermore, neurological damage, genetic alterations, and reproductive dysfunctions are major consequences of pesticides/toxicants that induce toxicity [40] Studies have recorded that aquaculture is rated as one of the crucial and critical sectors among other food-producing sectors in the world. Aquaculture plays a crucial role in meeting the demands of quality proteins [41,42]. Hence, monitoring the adverse impacts of various aquatic pollutants is of vital importance and necessary for minimizing deleterious effects on aquatic organisms and for optimal production. In earlier published literature, scanty information is available regarding the toxicity of thiram in freshwater fish (Pangasius). Therefore, this study was conducted to assess the genotoxicity, oxidative stress, and erythrocytic abnormalities in freshwater Pangasius fish exposed to a low level of thiram.

## Materials and methods

### Experimental design

This study was conducted following the guidelines devised by the bioethics committee of the Islamia University of Bahawalpur regarding the welfare and use of laboratory animals. Fifty Pangasius fish were placed in various glass aquariums and were acclimatized for two weeks under standard laboratory conditions to ensure their optimum health and to reduce any stress before the start of the experiment. After an acclimatization period, all the fish were arbitrarily divided into four groups, including one control/untreated group and three experimental treatment groups (T1, T2, and T3). The fish were placed in glass aquaria containing 80 L of water. The fish in groups T1, T2, and T3 were exposed to 5, 10, and 15 µg/L thiram mixed in aquarium water for 12 days. The concentrations of thiram were prepared by mixing in water, and the aquarium water was changed on daily basis for the renewal and maintenance of the actual concentrations of thiram.

### Animal ethics

All the study procedures were conducted following approval from the Animal Ethics Committee of the Office of Research Innovation and Commercialization, The Islamia University of Bahawalpur, and in compliance with the guidelines of the Institutional Animal Care and Use Committee (IACUC). The university-appointed research reviewers for monitoring of relevant ethical considerations confirmed that the study procedures adhered to established animal welfare standards. This study was also conducted following the guidelines devised by both the Bioethics Committee and Animal Ethics Committee of the Islamia University of Bahawalpur regarding the welfare and use of laboratory animals.

### Blood collection and erythrocytic changes

For blood collection, a sterile 26-gauge needle was used to collect the blood samples from the caudal vein of each fish reared in normal and treated groups. For estimation of morphological and nuclear alterations, a thin blood film from the blood sample collected without anticoagulant from each fish was prepared on a clean glass slide immediately after collection. All the prepared blood smears were then fixed with methanol and stained with Field Stain A and B for further analysis [43]. Changes (morphological and nuclear) in erythrocytes were recorded by examining the stained slides under a light microscope. The frequency of micronuclei, notched nuclei, lobed nuclei, condensed nuclei, and vacuolated nuclei, and different morphological alterations like microcytes, leptocytes, stomatocytes, and tear-shaped erythrocytes was observed in a total of 1000 erythrocytes of each fish. To determine the effects of thiram exposure, the percentage of abnormal cells in each group was calculated to reveal the genotoxic and morphological impact of thiram exposure [44].

### Methods of anesthesia and euthanasia

To minimize the stress before blood collection, all the fish were fasted for 10–12 h and were handled gently in a calm and shaded area. Before blood collection, fish were anesthetized to alleviate any kind of stress using clove oil (4 mg/L) according to our previous study [6]. The fish were monitored continuously until anesthesia was reached (loss of reflexes and absence of movement). After blood collection, the fish were placed in another tank containing the experimental water and euthanized using clove oil at higher concentrations (16 mg/L) to alleviate the stress. The fish were monitored continuously until euthanasia was reached, and death was confirmed by loss of reflexes. loss of consciousness and absence of movement.

### Oxidative stress and antioxidant profile

After blood collection, the fish in each group were carefully dissected for the separation of the gills and intestines. After collection, the gills and intestinal tissues were rinsed with ice-cold deionized water to get rid of blood and debris [45]. After that, these tissues were homogenized separately using chilled deionized water, and the homogenates were then centrifuged at 5,000 rpm for 5 min. The prepared samples were stored at −20°C for biochemical analysis. For estimation of CAT, POD, SOD, and GSH, approximately 100, 25, 20, and 100 μL tissue homogenate was used, respectively [46].

### Oxidative and antioxidant parameters

Different antioxidant enzymes were estimated according to earlier procedure, including SOD [47], GSH [48], POD [49], and CAT. The activity of oxidative stress parameters was measured according to a previously established protocol for TBARS and ROS [19] in homogenates of gills and intestine of pangasius fish with the help of a UV-spectrophotometer. The levels of glutathione (GSH) were quantified to evaluate the non-enzymatic antioxidant defense system.

### Histopathology

For histopathological analysis, the gills and intestinal tissues were immediately removed from all the fish at the time of dissection and were immediately preserved in 10% neutral buffered formaldehyde solution. After one week of fixation, approximately 4–5 µm-thick sections of gills and intestine were cut with the help of a microtome, dehydrated in ascending grades of ethanol, cleared in xylene, and stained using haematoxylin and eosin solutions [50].

### DNA damage assessment by comet assay

DNA damage assessment in isolated cells of the gills and intestine was determined using the comet assay technique. Low-melting agarose containing isolated cells of gill tissues and enterocytes was lysed, and the electrophoresis was performed under alkaline conditions. After that, the cells were neutralized and stained with ethidium bromide. The frequency of DNA damage was recorded and visualized using a fluorescence microscope. Quantification of DNA damage based on tail formation and nuclear materials fluorescing around the nucleus was carried out as previously reported [49].

### Statistical analysis

All data were expressed as mean ± standard error (SE). Statistical analysis was conducted using one-way analysis of variance (ANOVA) to determine the significant differences between the control and treatment groups. Tukey’s post hoc test, being a more acceptable, less objectionable, and widely used in statistical analysis, was applied for comparisons of results with significance set at *P ≤ 0.05*.

## Results

The results on frequency of microscopic observation of nuclear (Fig 1) and morphological (Fig 2) alterations in erythrocytes of Pangasius fish exposed to thiram at different concentrations revealed dose and time-dependent changes in the nucleus and morphology of the erythrocytes. A significant increase (*P ≤ 0.05*) in the percentage of erythrocytes with micronuclei was observed, particularly in the erythrocytes of pangasius fish at higher exposure (15 µg/L) levels at days 8 (1.73 ± 0.08) and 12 (3.45 ± 0.08) as compared to the control (0.38 ± 0.02) group. Significantly increased (*P ≤ 0.05*) frequency of formation of micronuclei (2.88 ± 0.09) was recorded in erythrocytes of group C at day 12 of the experiment. The frequency of notched nuclei in erythrocytes increased markedly in fish at the highest dose on day 12. Results exhibited an increased number of erythrocytes with lobed nuclei as compared to normal fish. The results exhibited noticeably higher frequency of erythrocytes with condensed nucleus, pear shape, vacuolated, and erythrocytes without nuclei (Figs 1-2) in pangasius fish at days 8 and 12 of the experiment. The result exhibited a noticeably increased percentage of erythrocytes without nuclei and erythrocyte nuclear remnants in fish exposed to a higher concentration (15 µg/L) of thiram at day 12 of the study (Fig 3). The percentage of erythrocytes with blabbed nuclei noticeably increased at day 12 in the highest exposure group.

**Fig 1:**
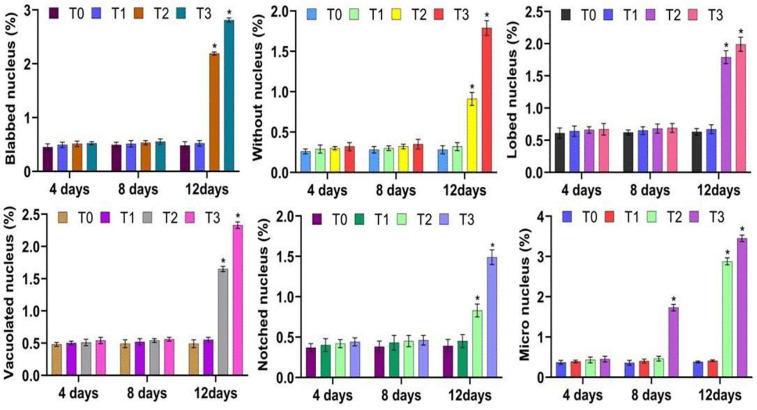
Various nuclear alterations observed in erythrocytes of fish given different doses of thiram. *P < 0.05.

**Fig 2:**
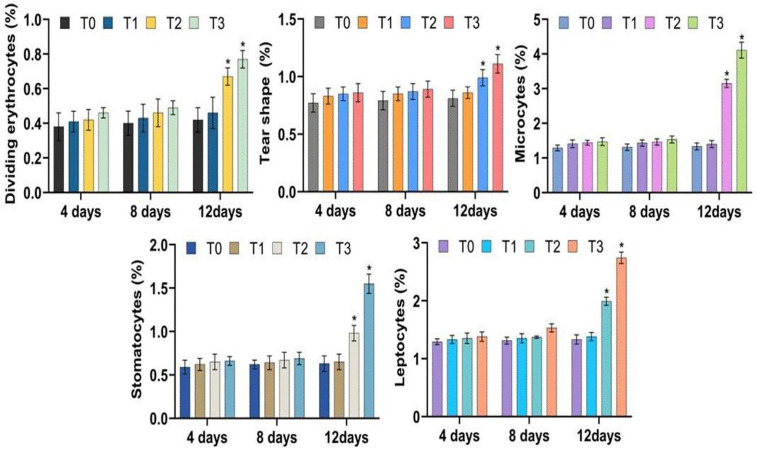
Various morphological alterations observed in erythrocytes of fish given different doses of thiram. *P < 0.05.

**Fig 3:**
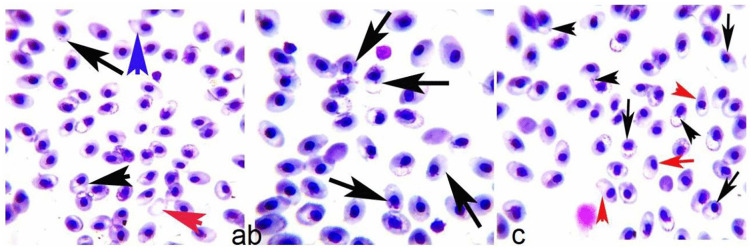
Blood smear (a-b) showing different morphological (pear shape erythrocyte; blue arrow head) and nuclear changes, cell with micronuclei (arrows), cell without nuclei (arrow head red), condensed nuclei (arrow head black) while c) showing pear shape (arrow heads red), vacuolated (arrow red), condensed nuclei (arrows black) and micronuclei (arrow heads black) in erythrocytes of pangasius fish exposed to thiram at high dose. Field Stain A & B; 1000X.

The results of morphological analysis demonstrated a significant increase in morphological alterations in erythrocytes of fish exposed to thiram at higher levels of exposure and in a time-dependent manner. A substantial increase in the percentage of dividing erythrocytes was observed, particularly in the fish reared in the higher exposure (15 µg/L) group on day 12 in comparison to the normal group. The results indicated remarkably higher percentages of leptocytes, stomatocytes, dividing erythrocytes, and tear-shaped erythrocytes in fish exposed to higher levels of thiram at day 12 of the research trial in comparison to normal fish.

### Oxidative stress and antioxidant enzymes

The results demonstrated significant alterations in the contents of antioxidant enzymes and oxidative stress biomarkers in the gills of fish exposed to thiram. A marked decrease (*P ≤ 0.05*) was recorded in the quantity of superoxide dismutase (SOD), peroxidase (POD), and catalase (CAT), indicating a suppression of the antioxidant defense system. The levels of reactive oxygen species (ROS) and thiobarbituric acid reactive substances (TBARS) were significantly (*P ≤ 0.05*) increased, correlating with an increased oxidative stress and lipid peroxidation (Fig 4).

**Fig 4:**
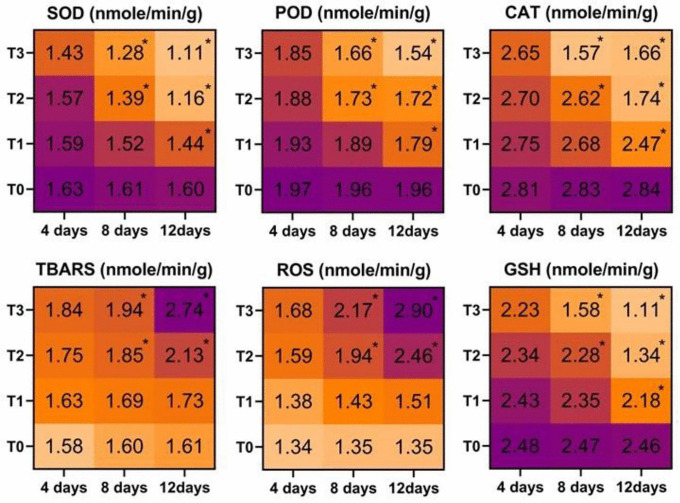
Antioxidant enzymes and oxidative stress parameters in gill tissues of Pangasius after exposure to various doses of thiram. *P < 0.05.

The results showed that the contents of SOD at days 8 and 12 of the trial remarkably decreased in the gills of fish reared at higher concentrations of thiram compared to normal fish. The progressive decline in the contents of key antioxidant enzymes, namely peroxidase (POD), catalase (CAT), and a non-enzymatic antioxidant glutathione (GSH), with the increase in doses and time of exposure, suggests suppression of the antioxidant capacity of the gills. The results on estimation of reactive oxygen species (ROS) showed a considerable increase in concentrations of ROS, especially at 12 days of exposure of fish in comparison to the normal group. The results on lipid peroxidation biomarker (thiobarbituric acid reactive substances; TBARS) revealed significantly increased contents at days 8 and 12 of the experiment in fish reared at higher doses of thiram. The decreased contents of antioxidant enzymes and increased amount of oxidative stress biomarkers were recorded in the intestine of fish exposed to thiram (Fig 5). The results showed noticeably lower contents of SOD, POD, CAT, and GSH with an increase in dose and time of exposure in intestinal tissues. The quantity of ROS and TBARS levels increased remarkably in intestinal tissues at days 8 and 12 in treated fish at a higher level of exposure. At histopathological examination, mild to moderate histo-architectural lesions were observed in various sections of the gills of fish at low doses throughout the experiment. Moderate (10 µg/L) to severe (15 µg/L) histopathological ailments, including degeneration of cartilaginous core, telangiectasia, degeneration and disruption of primary and secondary lamellae of gills, curling necrosis of lamellar epithelial cells, and uplifting of secondary lamellae were examined in fish treated with higher doses at day 12 of the experiment (Fig 6). At microscopic examination, degeneration of villi, necrosis of epithelial cells of intestinal villi, congestion, atrophy of villi, and sloughing of epithelium of villi were observed at day 12 of the exposure in fish exposed to higher levels (10 µg/L and 15 µg/L) of thiram.

**Fig 5:**
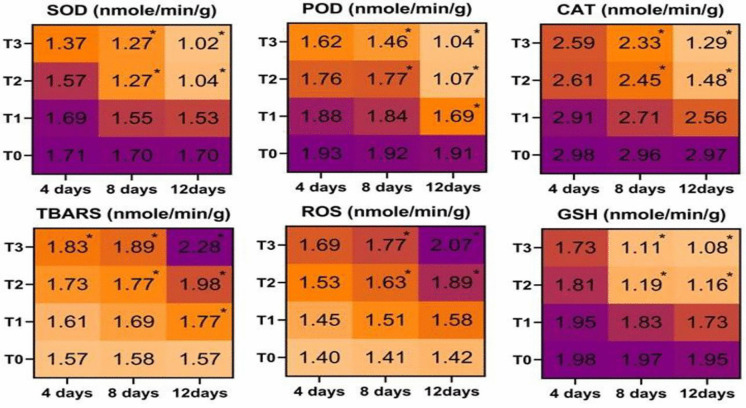
Antioxidant enzymes and oxidative stress parameters in intestinal tissues of Pangasius after exposure to various doses of thiram. *P < 0.05.

**Fig 6:**
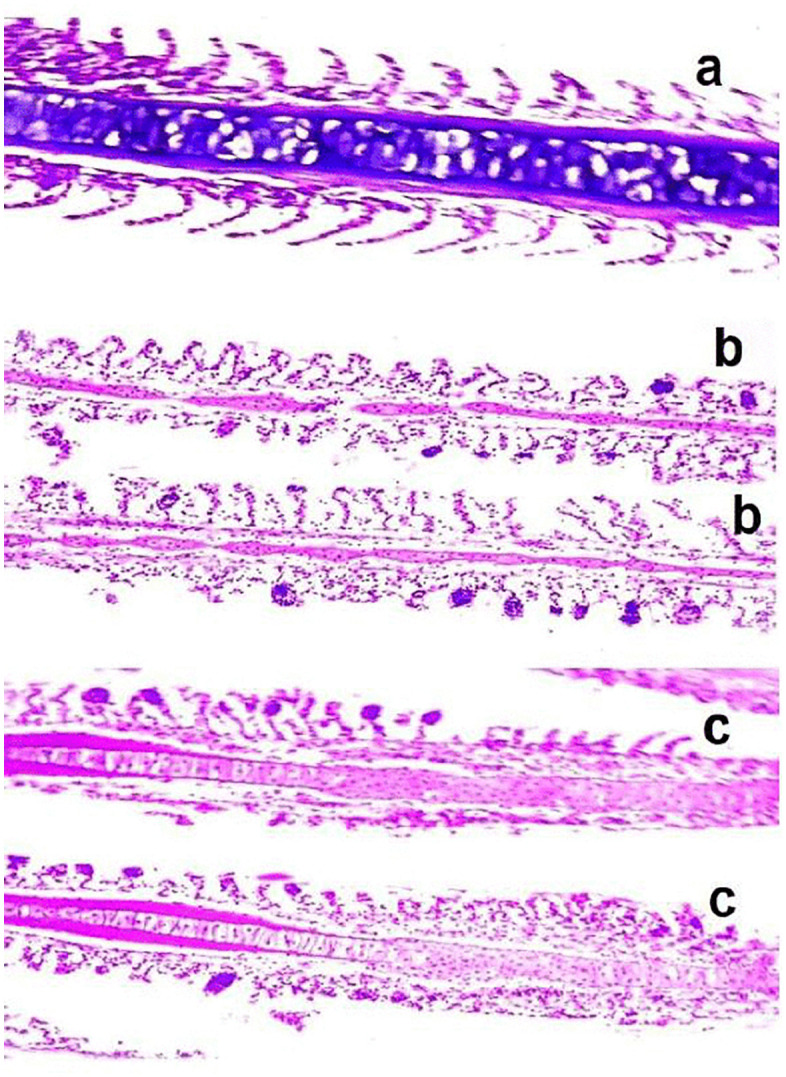
Photomicroscope of different sections of gills of treated fish showing normal histological arrangements (a), while b and c show different histopathological alterations in fish exposed to thiram at high doses. H & E stain; 400X.

### DNA damage

The results (Table 1) on the comet assay technique to determine the impairment of genetic material showed significant DNA damage in isolated cells of gills and intestinal tissues in a time and dose-dependent increase in terms of intensity of formation of the tail of the comet and fluorescence of DNA material around the nucleus. The extent of genomic instability significantly increased in isolated cells from gills at day 8 in fish of group T3 and at day 12 in groups T2-T3. The frequency of DNA damage in isolated enterocytes significantly increased (*P ≤ 0.05*) on days 8 and 12 in fish reared at a higher level of thiram in comparison to normal fish (Fig 7).

**Table 1:** Frequency of DNA damage by comet assay in isolated cells of intestine and gills of pangasius fish (*P < 0.05).

Parameters/ Days	Groups/Treatments			
	T0 (0.00 µg/L)	T1(5.0 µg/L)	T2(10.0 µg/L)	T3(15.0 µg/L)
Percentile rate of DNA damages in isolated cells of Gills				
4	1.23 ± 0.18	1.34 ± 0.16	1.40 ± 0.16	1.46 ± 0.13
8	1.24 ± 0.17	1.37 ± 0.18	1.43 ± 0.18	2.49 ± 0.14*
12	1.27 ± 0.15	1.41 ± 0.19	2.97 ± 0.15*	3.77 ± 0.16*
Percentile rate of DNA damage in isolated cells of the Intestine				
4	1.38 ± 0.13	1.43 ± 0.13	1.52 ± 0.13	1.55 ± 0.11
8	1.40 ± 0.11	1.45 ± 0.18	2.56 ± 0.14*	3.49 ± 0.14*
12	1.42 ± 0.15	1.49 ± 0.16	3.67 ± 0.15*	4.77 ± 0.15*

**Fig 7:**
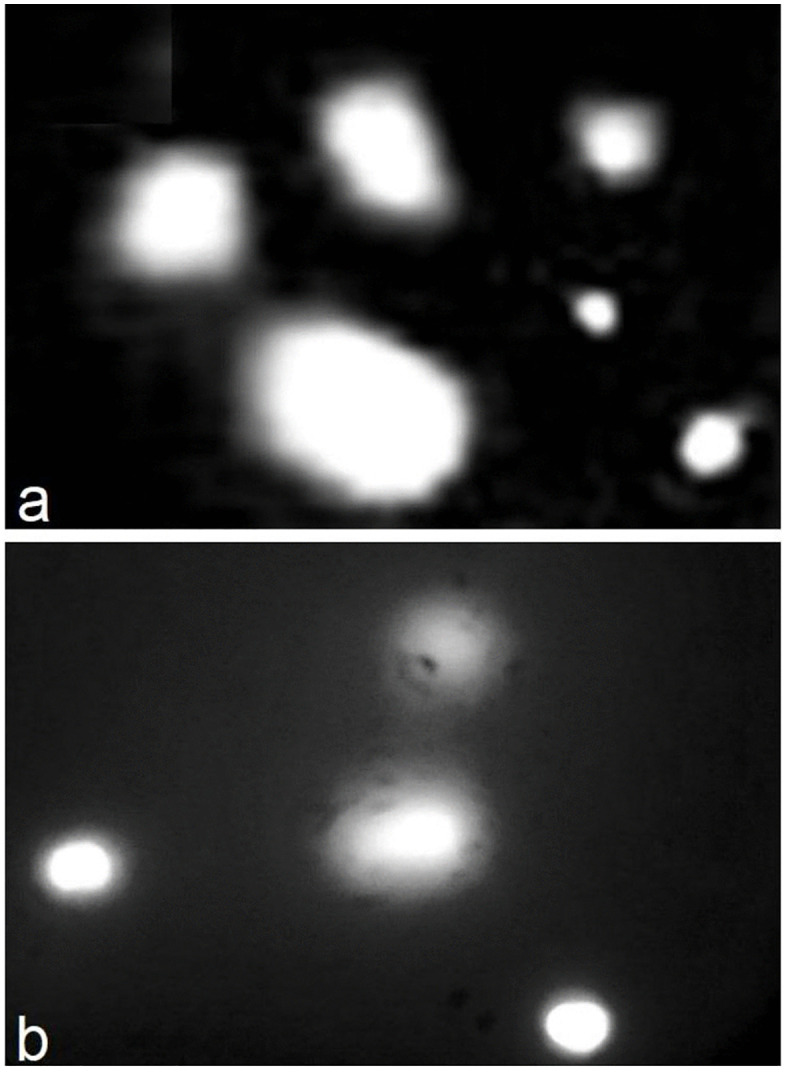
Photomicroscope showing DNA damage (tail formation and nuclear materials fluorescing around the nucleus) in isolated cells of the gills (a) and intestine (b) of fish exposed to thiram at higher doses. Ethidium bromide stain; 1000X.

## Discussion

Monitoring of inadequate applications of fungicides, herbicides, and pesticides in agriculture, veterinary practice, and public health management is of vital importance to mitigate the adverse toxic effects on aquatic and terrestrial life. It is recorded that the over-dependence of such types of chemicals poses serious threats and contaminates the aquatic ecosystem, ultimately influencing the health of the non-target organisms [7,40]. Studies have indicated that erythrocytes are considered reliable biomarkers for screening of oxidative stress induced by a variety of synthetic environmental compounds, such as pesticides and insecticides. Erythrocytes reflect almost all physical and chemical changes in various organisms exposed to different contaminants/pollutants [51]. The significantly increased erythrocytic abnormalities (micronuclei, notched nuclei, lobed nuclei, and tear-shaped erythrocytes) in Pangasius fish in this study might be due to the over-release of free radicals, causing damage to cellular proteins. Micronucleus formation is a biomarker of genotoxicity, and it is usually associated with oxidative stress and DNA damage [45]. The significantly increased percentile rate of nuclear and morphological abnormalities in the present study in fish exposed to higher concentrations of thiram might also be linked to induction of abnormal apoptotic changes. Similar erythrocytic changes in freshwater fish subjected to broad-spectrum fungicide (azoxystrobin) have also been observed [44]. Previous studies have reported that pesticides and insecticides induce oxidative stress and directly damages nuclear materials including DNA, proteins and cell membranes in exposed organisms [34,52]. The nuclear and morphological changes observed in this study may be related to interference of thiram with mitotic process which has been shown to induce chromosomal instability and micronuclei formation [53]. The tear-shaped and other morphologically alterations in erythrocytes are suggestive of disruptions of cytoskeletal proteins caused by oxidative stress. Similar erythrocytic nuclear abnormalities in Nile tilapia exposed to profenofos have also been recorded [54].

Significantly higher contents of biomarkers of oxidative stress in Pangasius fish exposed to thiram were evident at higher levels of the fungicide in the gills and intestinal tissues. Antioxidant enzymes, including superoxide dismutase (SOD), peroxidase (POD), and catalase (CAT), play an important role in neutralizing ROS and maintaining the cellular redox balance [23,29]. Similar to our results on antioxidant enzymes, significantly lower antioxidant activities were recorded in fish (*Labeo rohita*) due to glyphosate-induced oxidative stress [6]. Previously, hemato-biochemical and histopathological alterations in *Labeo rohita* fish treated with pyriproxyfen due to oxidative stress have been examined [45]. It has been investigated that the lower contents of antioxidant enzymes are linked to excessive production of ROS, which led to the loss of their activity [47,55]. The higher contents of thiobarbituric acid reactive substance (TBARS) in the gills and intestinal tissues in treated fish in this study are suggestive of lipid peroxidation leading to DNA damage. The significantly reduced contents of intracellular enzyme (GSH) and various antioxidant enzymes (SOD, POD, and CAT) might be linked with increased production of MDA, causing lipid membrane peroxidation [25]. In addition, the substantial reduction in values of SOD, POD, CAT, and GSH, while increased contents of ROS and TBARS in fish could be due to disruption of normal physiological mechanisms (redox homeostasis) in terms of upregulation of signaling pathways (HIF-1/VEGF, Rap1-PI3K/AKT and Ras-MAPK) in treated fish [9]. Moreover, the depletion of antioxidant enzymes, erythrocytic ailments, and histopathological alteration in Pangasius fish could be due to protonation-induced Mus GPR4 and Xenopus GPR4 activation [56]. The significantly increased levels of TBARS in Oreochromis niloticus [57] and cockerels have been reported due to exposure to thiamethoxam, causing severe damage at the nucleotide level of DNA. Glutathione (GSH), which is also a key non-enzymatic antioxidant, was also highly depleted in the tissues of the gills and intestine of pangasius fish due to exposure to thiram, demonstrating the intense oxidative stress. The lower contents of GSH in treated fish might be because of counteractions of the ROS in recovery of the damaged molecules during oxidation [58]. Findings of reduced GSH in this study are in line with previous reports [19], where GSH levels were found to be reduced in common carp due to oxidative and antioxidative imbalance. GSH may also be depleted due to utilization in neutralization of ROS or regeneration of other antioxidants, vitamin C, and vitamin E [59]. Furthermore, the sub-acute oxidative stress elucidated in this study may also disrupt GSH synthesis, escalating GSH depletion [60].

Elevated ROS levels observed in this study are considered a central mechanism of thiram-induced toxicity. ROS overproduction can damage the vital cell components such as lipids, proteins, and nucleic acids, resulting in cell dysfunction leading to DNA damage and histoarchitectural changes [20,61–63]. The histopathological changes in gills and intestinal tissues might be related to oxidative stress-mediated toxicity in fish exposed to thiram at high doses. Previously, intestinal atrophy, necrosis of intestinal mucosa, and sub-mucosal hemorrhages were observed in O. niloticus fish obtained from an environmentally contaminated area [39,64] Histopathological alterations observed in the gills of pangasius fish in this experimental study, including aneurysm, lamellar fusion, congestion of cartilaginous core, and necrosis of lamellar epithelial cells, have also been recorded in various species of freshwater fish exposed to toxicants.

Notably, tail length, tail intensity, and tail moment were all increased significantly in isolated cells of gills and intestinal tissues of pangasius fish, signifying extensive DNA fragmentation and genomic instability. These results are consistent with previous studies [34,39,65,66], where similar increases in DNA damage in fish were recorded. The DNA damage in isolated cells might be related to ROS-induced oxidative stress, responsible for oxidizing DNA bases, inducing strand breaks, and disrupting the structural integrity of the genome [51,67]. DNA damage observed in fish due to exposure to thiram may also correlate with depletion of antioxidant enzymes in fish [6,68]. Thiram metabolites can directly induce DNA damage, highlighting their genotoxicity [60]. The comet assay sensitivity to identify DNA fragmentation is known as a critical validation of the important effect of thiram on the genomic stability of aquatic organisms. Disrupted DNA repair mechanisms during oxidative stress also increase the genotoxic effects [60,69]. The results on nuclear and morphological alterations in erythrocytes, significantly higher contents of oxidative stress (ROS and TBARS), depletion of antioxidant enzymes, DNA damage, and histopathological changes in Pangasius fish are suggestive of physiological disorders induced by oxidative stress. The histopathological alteration in gills and intestinal tissues of pangasius fish at higher doses might be due to mitochondrial dysfunctions and disruption of cellular membranes [45,70]. Moreover, increased DNA damage in isolated cells of gills and intestinal tissues, along with histopathological lesions in our study in pangasius fish, could also be linked to the release of cytochrome C due to overgeneration of ROS, leading to damage to mitochondrial membrane potential [71]. The histopathological lesions in fish due to exposure to thiram in the gills of common carp [39] and different visceral organs (heart, kidneys, liver, and brain) of labeo rohita have also been observed [34]. This study could not determine the molecular pathways to know the exact mechanisms of induction of toxicity in Pangasius fish. Therefore, future studies are suggested to explore these mechanisms.

## Conclusion

The study demonstrated that thiram exposure induces significant oxidative stress, genotoxicity, and erythrocytic alterations in fish, highlighting its detrimental impacts on aquatic organisms. Increased oxidative stress biomarkers in gills and intestines, while marked suppression of antioxidant defenses in gills and intestines was observed due to sub-acute exposure, as evidenced by higher contents of ROS, TBARS, and lower contents of SOD, POD, CAT, and GSH. Thin blood film/smear analysis revealed significantly higher nuclear and morphological abnormalities in erythrocytes of fish, including micronuclei and abnormal shapes, suggestive of genotoxic and cytotoxic effects. In addition, increased frequency of DNA damage in isolated cells of gills and intestine, as determined by the comet assay, unveiled genotoxic potential. Hence, for sustainable growth of aquaculture and protection of aquatic organisms, large-scale epidemiological studies focusing on chronic exposure and mitigation strategies are recommended to safeguard aquatic agricultural practices.

## Supporting information

S1 Data (XLSX)
